# Acridine Derivatives as Antifungal and Antivirulence Agents Against *Candida albicans*

**DOI:** 10.3390/ijms26157228

**Published:** 2025-07-25

**Authors:** Amra Yunus, Oluwatosin Oluwaseun Faleye, Jin-Hyung Lee, Jintae Lee

**Affiliations:** School of Chemical Engineering, Yeungnam University, Gyeongsan 38541, Republic of Korea; amrayunus321@yu.ac.kr (A.Y.); oluwatosin@yu.ac.kr (O.O.F.)

**Keywords:** acridine derivatives, antifungal, antivirulence, *Candida albicans*

## Abstract

*Candida albicans* is a clinically important fungal pathogen capable of causing both superficial and systemic infections, particularly in immunocompromised individuals. A key factor contributing to its pathogenicity is its ability to form biofilms, structured microbial communities that confer significant resistance to conventional antifungal therapies. Addressing this challenge, we explored the antivirulence potential of acridine derivatives, a class of heterocyclic aromatic compounds known for their diverse biological activities, including antimicrobial, antitumor, and antiparasitic properties. In this study, a series of acridine derivatives was screened against *C. albicans* biofilms, revealing notable inhibitory activity and highlighting their potential as scaffolds for the development of novel antifungal agents. Among the tested compounds, acridine-4-carboxylic acid demonstrated the most promising activity, significantly inhibiting the biofilm formation at 10 µg/mL without affecting planktonic cell growth, and with a minimum inhibitory concentration (MIC) of 60 µg/mL. Furthermore, it attenuated filamentation and cell aggregation in a fluconazole-resistant *C. albicans* strain. Toxicity assessments using *Caenorhabditis elegans* and plant models supported its low-toxicity profile. These findings highlight the potential of acridine-based scaffolds, particularly acridine-4-carboxylic acid, as lead structures for the development of therapeutics targeting both fungal growth and biofilm formation in *Candida albicans* infections.

## 1. Introduction

Fungal infections caused by *Candida albicans* are increasingly recognized as a major public health challenge worldwide [1,2,3]. As a natural member of the human microbiota, *C. albicans* typically resides harmlessly on mucosal surfaces [4]. However, in immunocompromised individuals, it can shift to a pathogenic state, leading to a wide range of infection, from superficial conditions such as oral thrush and vulvovaginal candidiasis to severe, systemic infections like candidemia, endophthalmitis, osteomyelitis and septic arthritis, and meningitis and central nervous system infections [5,6,7,8,9]. The ability of *C. albicans* to cause disease is closely linked to its versatile virulence factors, including morphological transitions between yeast and hyphal forms, which are hallmarks of virulence and immune evasion in *C. albicans* biofilms [10], the secretion of tissue-damaging enzymes, and a robust quorum-sensing network that facilitates biofilm formation and immune evasion [11,12].

A critical aspect of *C. albicans* pathogenicity is its ability to form biofilms, particularly on abiotic surfaces like catheters, prosthetic valves, and implants [13,14]. These biofilms are dense microbial communities embedded in an extracellular polysaccharide matrix that confers resistance to antifungal agents and protection from host immune responses. As a result, biofilm-associated infections are often recalcitrant, persistent, and associated with significantly increased morbidity and mortality [15].

Despite the availability of antifungal drugs such as azoles, echinocandins, and polyenes, clinical management of candidiasis is hindered by rising drug resistance, toxicity, and reduced efficacy against biofilm-associated cells [16,17]. The limitations of current therapies underscore the urgent need for novel antifungal agents with alternative mechanisms of action, particularly those targeting biofilm integrity and formation.

In recent years, acridine-based compounds have gained attention for their antimicrobial potential. Acridines, known for their planar tricyclic structure, can intercalate with DNA and disrupt vital cellular processes. Certain acridine derivatives, such as 9-anilinoacridine and 1-nitro-9-aminoacridine, have exhibited promising activity against *C. albicans*, including inhibition of biofilm formation, hyphal development, and virulence expression [18]. Their structure also allows fine-tuning of functional groups to enhance antifungal specificity and reduce toxicity.

In this study, we investigated the antifungal and biofilm inhibitory activity of structurally related acridine-based compounds against a fluconazole-resistant *C. albicans* strain. Thirteen acridine compounds were screened for their ability to inhibit both planktonic cell growth and biofilm formation of *C. albicans*. The most active acridine-4-carboxylic acids were further assessed for their effects on virulent traits such as hyphal formation, filamentous protrusion, and cell aggregation. Additionally, their toxicity profiles were preliminarily evaluated using *Caenorhabditis elegans* and seed germination models, complemented by in silico ADME-Tox (Absorption, Distribution, Metabolism, Excretion and Toxicity) analysis. These findings aim to establish acridine-4-carboxylic acid as a promising scaffold in the development of novel antifungal therapies against *Candida*-related infections.

## 2. Results

### 2.1. Effects of Acridine Derivatives on Biofilm Formation and Planktonic Cells of C. albicans

**Figure 1:**
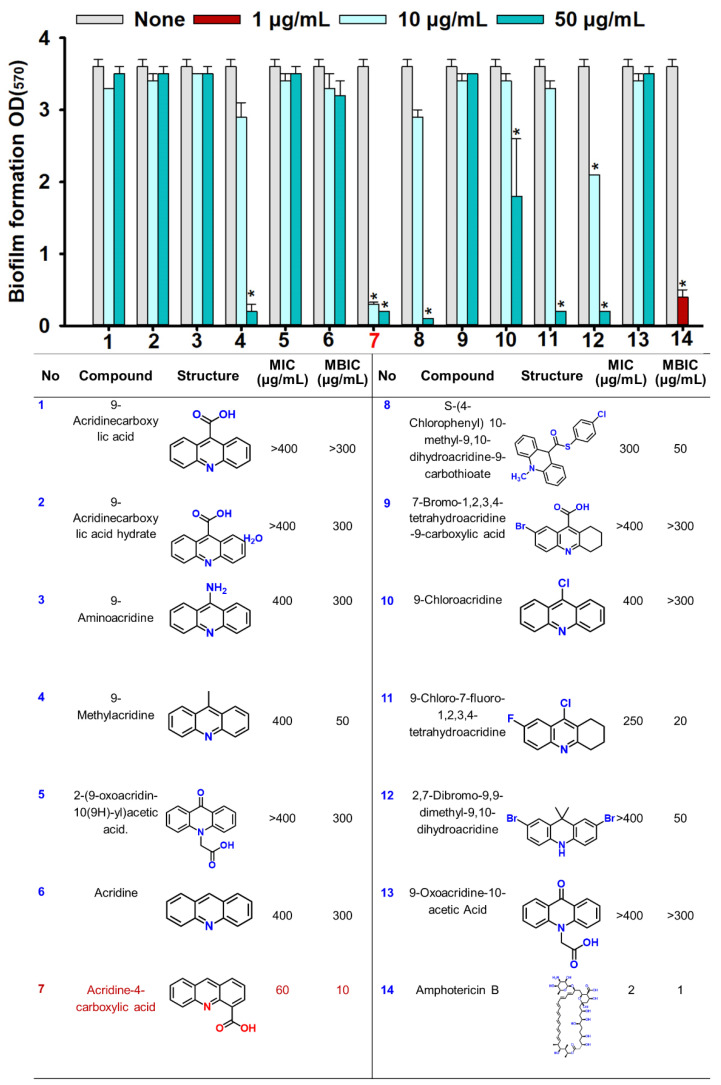
The biofilm inhibitory and antifungal screening of acridine derivatives. Biofilm formation by *C. albicans* DAY185 with acridine derivatives at 10 and 50 µg/mL in 96-well polystyrene plates after 24 h culture. Asterisk (*) indicates significant differences of biofilm formation (*p* < 0.05), and error bars display the standard deviation. The listed numbers correspond to the chemical names and their respective structures. Compound 7 highlighted in red in the table was identified as the most active. Amphotericin B was used as a positive control.

**Figure 2:**
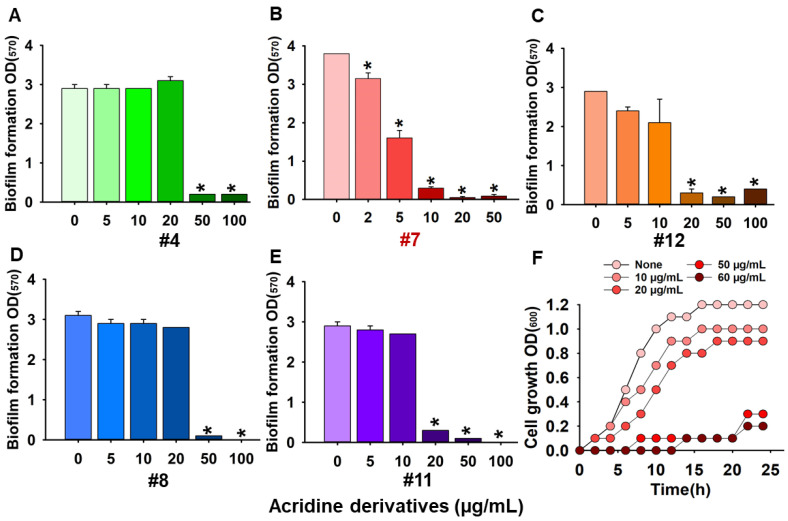
Dose-dependent screening of acridine derivatives #4: 9-methylacridine (**A**), #7: acridine-4-carboxylic acid (**B**), #12: 2,7-dibromo-9,9-dimethyl-9,10-dihydroacridine (**C**), #8: S-(4-chlorophenyl) 10-methyl-9,10-dihydroacridine-9-carbothionate (**D**), #11: 9-chloro-7-fluoro-1,2,3,4-tetrahydroacridine (**E**), and planktonic cell growth in the presence of #7: acridine-4-carboxylic acid (**F**). Asterisk (*) denotes a significant difference at p < 0.05 and error bars represent the standard deviation.2.2. Acridine Derivative Inhibits Hyphal Formation, Cell Aggregation, Biofilm Structure, and Metabolic Activity.

The biofilm inhibitory activities of 13 acridine derivatives were initially assessed against *Candida albicans* DAY185, using amphotericin B as a positive control. Among the tested compounds, acridine-4-carboxylic acid (compound #7) emerged as the most potent inhibitor of biofilm formation, achieving 86% inhibition at a minimum biofilm inhibitory concentration (MBIC) of 10 µg/mL. Compound #11 (9-chloro-7-fluoro-1,2,3,4-tetrahydroacridine) followed closely, exhibiting 91% inhibition at 20 µg/mL. Additional derivatives including compounds #4, #8, and #12 demonstrated strong antibiofilm efficacy, achieving 93.1%, 93.5%, and 89.9% inhibition, respectively, at 50 µg/mL (Figure 1). To further validate their inhibitory potential, the five most active derivatives (#4, #7, #8, #11, and #12) were dose-dependently assessed (Figure 2). Expectedly, acridine-4-carboxylic acid exhibited dose-dependent inhibition of biofilm formation, with significant reduction evident at 5 µg/mL and complete inhibition achieved at concentrations ≥10 µg/mL (Figure 2B). Compounds #11 and #12 also showed substantial biofilm inhibition, with strong effects observed between 20 and 100 µg/mL (Figure 2C,E), while #4 and #8 caused >80% inhibition at 50 µg/mL (Figure 2A,D). These findings identified compound #7 as the most effective biofilm preventive agent and prompted its selection for further analysis.

To explore whether acridine-4-carboxylic acid could disrupt mature biofilms, a dispersal assay was conducted on established *C. albicans* biofilms. At 4× MIC, the compound achieved only moderate dispersal effects, with 27% biofilm reduction. These results suggest that while acridine-4-carboxylic acid is a robust inhibitor of biofilm formation, its capacity to disrupt preformed biofilms is limited.

Furthermore, the antifungal activity of acridine-4-carboxylic acid was evaluated against *C. albicans* DAY185. Among the tested compounds, only compound #7 displayed measurable antifungal activity against planktonic cells, with a minimum inhibitory concentration (MIC) of 60 µg/mL. In contrast, other derivatives had MIC values exceeding 250 µg/mL (Figure 1). Growth curve analysis confirmed that acridine-4-carboxylic acid inhibited planktonic proliferation in a dose-dependent manner, with complete suppression observed at its MIC (Figure 2F). To assess its broader antifungal spectrum, MIC values of acridine-4-carboxylic acid were determined against eight clinically relevant *Candida* strains. The compound demonstrated differential activity across species. Specifically, *C. albicans* ATCC 10231 and *C. glabrata* ATCC 2001 exhibited MICs of 350 µg/mL, while *C. glabrata* KCCM 50701 showed a MIC of 250 µg/mL. *C. parapsilosis* strains showed MICs ranging from 200 to 300 µg/mL, while *Candida auris* displayed better susceptibility, with MICs of 100 µg/mL (KCTC 17809) and 150 µg/mL (KCTC 17810). These findings demonstrate that acridine-4-carboxylic acid exhibits selective antifungal activity, with the greatest efficacy against fluconazole-resistant *C. albicans* DAY185 and multidrug-resistant *C. auris* strains.

**Figure 3:**
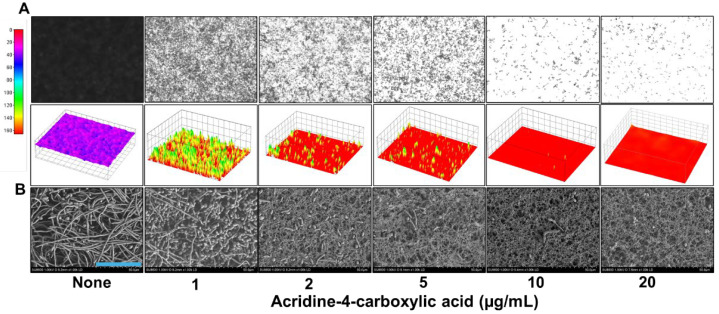
Microscopic imaging of *C. albicans* DAY185 biofilms treated with acridine-4-carboxylic acid (**A**). SEM analysis shows *C. albicans* DAY185 biofilms exposed to acridinie-4-carboxylic acid (**B**). Blue scale bar represents 50 µm.

**Figure 4:**
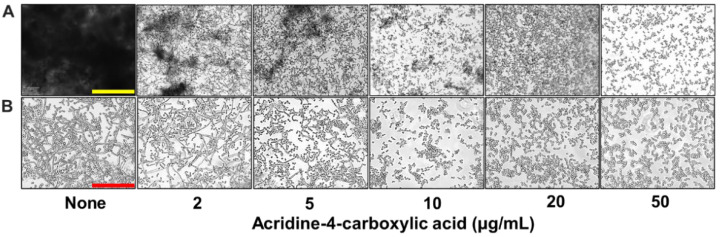
Inhibition of virulence factor production by acridine-4-carboxylic acid. Cell aggregation (**A**) and hyphal formation (**B**). Yellow and red scale bars represent 200 and 100 µm, respectively.

To assess the impact of acridine-4-carboxylic acid on these virulence traits, a series of microscopic and imaging analyses were conducted. Live-cell imaging revealed that biofilm formation decreased progressively with increasing concentrations of acridine-4-carboxylic acid (Figure 3A). At 2 µg/mL, a substantial reduction in biofilm was observed, while near complete inhibition occurred at 10 µg/mL, as visualized through both 2D and 3D color-coded biofilm surface plots. Scanning electron microscopy (SEM) analysis provided high-resolution confirmation of these inhibitory effects (Figure 3B). Untreated controls showed dense networks of elongated hyphae intertwined with yeast cells, characteristic of mature biofilms. In contrast, biofilms treated with acridine-4-carboxylic acid exhibited a strong reduction in hyphae. Bright-field microscopy of liquid cultures also demonstrated that acridine-4-carboxylic acid markedly reduced cell aggregation (Figure 4A) and hyphal development (Figure 4B) at 10–20 µg/mL, and compared to untreated controls, treated samples displayed fewer filamentous structures and predominantly yeast-form cells, indicating a blockage in the morphogenetic transition.

**Figure 5:**
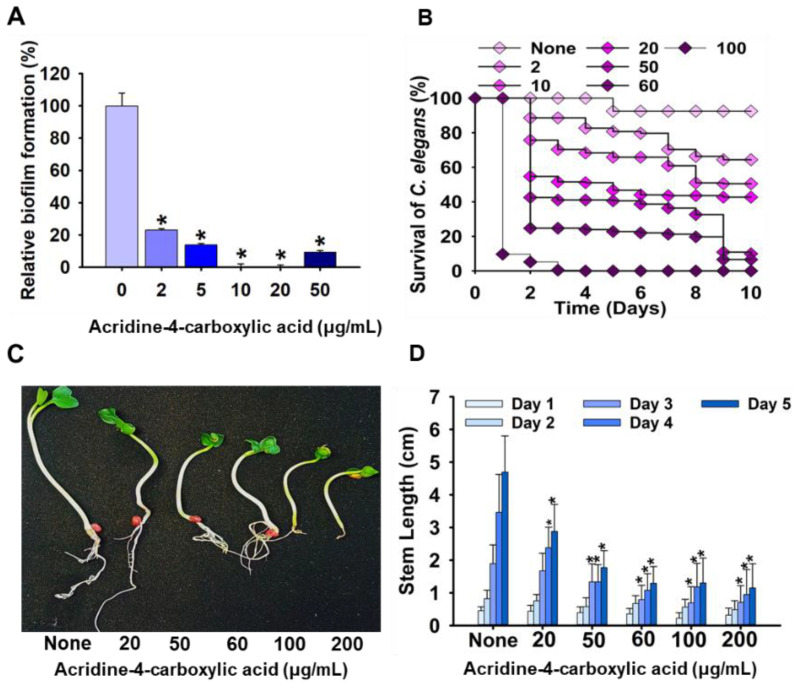
Effect of acridine-4-carboxylic acid on early-stage biofilm metabolic activity, assessed by the XTT reduction assay (**A**); toxicity study of acridine-4-carboxylic acid in nematode (**B**); and plant models (**C**,**D**). Asterisk (*) denotes a significant difference at *p* < 0.05 and error bars represent the standard deviation.

Acridine-4-carboxylic acid markedly suppressed early-stage biofilm metabolic activity in a dose-dependent manner, as demonstrated by the XTT (2,3-Bis(2-methoxy-4-nitro-5-sulfophenyl)-5-[(phenylamino)carbonyl]-2H-tetrazolium hydroxide) assay (Figure 5A). Treatment with 5–50 µg/mL of acridine-4-carboxylic acid led to a greater than 90% reduction in metabolic activity compared to the untreated control, indicating a substantial inhibitory effect on viable biofilm-associated cells. This metabolic inhibition is consistent with the observed reductions in hyphal development, cell aggregation, and biofilm formation, as confirmed by the crystal violet assay. These findings suggest that acridine-4-carboxylic acid disrupts the morphogenetic transition and early biofilm formation in *C. albicans*. Collectively, these findings demonstrate that acridine-4-carboxylic acid not only inhibits biofilm formation but also effectively suppresses *C. albicans* hyphal development and cell aggregation from 10 µg/mL, reinforcing its potential as a targeted antifungal agent against biofilm-associated infections.

### 2.2. Effect of Acridine-4-carboxylic Acid on Plant Growth and Nematode Model

To evaluate the toxicity of acridine-4-carboxylic acid, its impact on plant development was investigated using a radish seed germination model. Seedlings of *Raphanus sativus* were exposed to acridine-4-carboxylic acid at concentrations ranging from 20 to 200 µg/mL, and stem length was recorded over a five-day period. The results demonstrated a concentration-dependent inhibition of shoot elongation, with the most significant reductions observed at ≥60 µg/mL (Figure 5C,D). At 20 µg/mL, only mild growth inhibition was observed compared to the untreated control, whereas doses of 100 and 200 µg/mL resulted in visibly stunted growth and reduced leaf expansion. The cytotoxicity of acridine-4-carboxylic acid was further evaluated using a *Caenorhabditis elegans* nematode survival assay (Figure 5B). At lower concentrations (2–10 µg/mL), survival rates remained high, exceeding 85%, indicating minimal toxicity. However, a marked, dose-dependent decline in viability was observed at higher concentrations. At 50 µg/mL, survival dropped to below 50% by Day 6, while exposure to 100 µg/mL resulted in rapid mortality, with nearly all nematodes dead by Day 4. These results align with the observed phytotoxicity profile (Figure 5C,D), demonstrating that acridine-4-carboxylic acid is well tolerated at concentrations effective for biofilm inhibition (≤20 µg/mL) but exhibits significant toxicity at doses approaching or exceeding its MIC.

### 2.3. In Silico Absorption, Distribution, Metabolism, Excretion (ADME) Profiling of Acridine Derivatives

Three acridine derivatives, namely, acridine, acridine-4-carboxylic acid, and 2,7-dibromo-9,9-dimethyl-9,10-dihydroacridine, were evaluated using in silico models to assess their pharmacokinetic, toxicity, and drug-likeness profiles. Acridine-4-carboxylic acid emerged as a promising candidate, exhibiting no violations of key drug-likeness filters (Lipinski, Veber, Muegge) and demonstrating a balanced ADMET profile. It showed moderate gastrointestinal absorption, acceptable Caco-2 permeability (30.3 nm/s), and low lipophilicity (LogP 3.0; miLogP 2.69), favoring oral bioavailability and reducing the likelihood of off-target accumulation. The compound also demonstrated moderate blood–brain barrier permeability (2.27), suggesting potential utility in treating invasive fungal infections affecting the central nervous system [19,20]. From a metabolic standpoint, acridine-4-carboxylic acid was non-inhibitory toward key CYP450 isoforms (CYP1A2, CYP3A4) and showed no pharmacological activity against GPCRs, ion channels, or protease targets, minimizing the risk of off-target effects. It was also non-substrate and non-inhibitory to P-glycoprotein, indicating a low propensity for efflux-mediated resistance. Its high plasma protein binding (~93%) further supports the potential for extended systemic circulation. In contrast, the third derivative exhibited poor solubility, high lipophilicity (LogP > 15), and P-gp inhibition, raising concerns over bioavailability, resistance development, and environmental persistence. All three derivatives displayed medium hERG inhibition potential, moderate toxicity, and positive mouse carcinogenicity with negative findings in rats, indicating species-specific toxicological responses. Environmental safety predictions for acridine-4-carboxylic acid revealed low acute toxicity to non-target aquatic organisms, including algae (0.09 µg/L), medaka fish (0.017 µg/L), and minnow (0.015 µg/L), suggesting a potentially low ecological risk. Complementing these data, phytotoxicity testing in a radish (*R. sativus*) seed germination model revealed only mild growth suppression at 20 µg/mL, with significant inhibition of shoot and root development observed at concentrations ≥ 60 µg/mL.

## 3. Discussion

The emergence of antifungal resistance in *Candida albicans*, especially in biofilm-associated infections, presents a significant clinical challenge. Biofilms confer up to a 1000-fold increase in resistance to antifungals like fluconazole [21], primarily due to the protective extracellular matrix that impedes drug penetration and promotes immune evasion. These factors contribute to persistent infections, treatment failure, and higher mortality [22,23].

In this study, we investigated the antifungal and antivirulence properties of acridine derivatives against a fluconazole-resistant *C. albicans* strain. Among the tested compounds, acridine-4-carboxylic acid exhibited the most promising activity, significantly inhibiting biofilm formation at sub-inhibitory concentrations (MBIC = 10 µg/mL), without affecting planktonic growth (MIC = 60 µg/mL). This was followed by 9-chloro-7-fluoro-1,2,3,4-tetrahydroacridine with an MBIC of 20 µg/mL (Figure 1). This selective antibiofilm action is consistent with previous studies on ethacridine, an acridine derivative that inhibited biofilm formation without compromising *C. albicans* planktonic viability [24]. Also, acridine-based photosensitizers demonstrated similar efficacy in reducing biofilm formation by *Staphylococcus aureus* [25]. Targeting biofilm formation rather than cell viability is a recognized strategy to mitigate antifungal resistance [26,27]. Hence, the potent sub-MIC activity of acridine-4-carboxylic acid suggests a reduced risk of resistance development by minimizing selective pressure (Figure 2B,F). Structural analysis suggests that the carboxylic acid moiety in acridine-4-carboxylic acid increases hydrophilicity and potentially promotes selective targeting of biofilm-forming cells [28]. In contrast, 9-chloro-7-fluoro-1,2,3,4-tetrahydroacridine, with halogen substitutions and partial saturation (tetrahydro) of the acridine ring, may increase lipophilicity and influence membrane interactions [29]. These structural differences may underlie the observed variation in MBIC values (Figure 1), indicating the critical role of polarity and electronic distribution in modulating antibiofilm activity.

Moreover, the biofilm inhibitory effect visualized by live imaging microscopy and SEM confirmed a reduction in surface-attached biofilm and its structural integrity. Also, SEM images of treated *C. albicans* cells displayed a marked decrease in hyphal density and a predominance of yeast-form cells, suggesting that acridine-4-carboxylic acid interferes with hyphal morphogenesis (Figure 3A,B). This is consistent with previous reports that 9-(2′-hydroxyethylamino)-1-nitroacridine and 9-(2′-hydroxyethylamino)-4-methyl-1-nitroacridine reduced hyphal development and biofilm formation in *C. albicans* rather than direct fungicidal activity [30]. This interference with hyphal morphogenesis is significant, as hyphal formation is critical for *C. albicans* virulence and biofilm maturation [31,32]. Similarly, acridine-4-carboxylic acid effectively reduced cell aggregation and filamentation from concentrations as low as 10 µg/mL (Figure 4A,B). These findings align with prior research emphasizing the therapeutic relevance of suppressing fungal virulence while minimizing growth inhibition and resistance development [33,34,35]. Similar dual effects on biofilm formation and morphogenesis have been reported for 5-hydroxymethyl-2-furaldehyde and Mannich bases, the latter acting through the Ras-cAMP-PKA pathway [36,37].

Additionally, the combined inhibition of cell aggregation, hyphal development, and metabolic activity indicates that acridine-4-carboxylic acid targets multiple stages of biofilm establishment (Figure 4 and Figure 5A). Since hyphal morphogenesis is essential for biofilm maturation and structural integrity in *Candida albicans* [38], the suppression of filamentation likely contributes to the observed reductions in both biomass and metabolic viability. Complementary XTT assays demonstrated a >90% reduction in biofilm metabolic activity at 5–50 µg/mL, consistent with the reductions in biomass and hyphal development observed microscopically. This confirms that acridine-4-carboxylic acid inhibits both biofilm formation and the viability of biofilm-associated cells, supporting its role as an early-stage biofilm inhibitor and antivirulence agent. While our phenotypic analyses suggest potential interference with key morphogenic pathways, molecular-level investigation through transcriptomic or qRT-PCR analyses was not conducted in the present study. Future investigations involving gene expression profiling could provide deeper insights into the modulation of virulence-associated genes and the underlying mechanisms of action.

Although the primary focus of this study is the biofilm preventive potential of acridine-4-carboxylic acid, we also assessed its ability to eradicate established biofilms at MIC, 2× MIC, and 4× MIC. The result revealed only modest biofilm reduction, even at concentrations 12 to 24 times higher than those required to inhibit biofilm formation. This observation reinforces established reports that biofilm eradication is substantially more challenging than prevention. Nonetheless, the focus on biofilm prevention is therapeutically relevant, as inhibiting early-stage biofilm development such as adhesion and EPS production reduces the likelihood of resistance evolution by avoiding selective pressure on growth [39,40]. Taken together, these findings support the potential of acridine-4-carboxylic acid as a promising antivirulence candidate for the prophylactic control of *Candida* biofilms.

Furthermore, acridine-4-carboxylic acid demonstrated antifungal activity against eight additional clinically relevant *Candida* strains. Notably, it exhibited enhanced efficacy against two multidrug-resistant *Candida auris* isolates, with MICs ranging from 100 to 150 µg/mL, suggesting potential effectiveness against species associated with clinical resilience and high treatment failure rates. Although the MIC values for most strains were higher compared to the 60 µg/mL observed for *C. albicans* DAY185, a fluconazole-resistant strain, the data collectively point to a strain-dependent antifungal effect. This specificity may reflect differences in membrane composition, efflux activity, or virulence-associated regulatory pathways among *Candida* species. From a therapeutic standpoint, such selective potency is particularly promising for targeting drug-resistant or virulent isolates, such as *C. auris* and *C. albicans* DAY185, where conventional antifungals often fail. These observations suggest the potential of acridine-4-carboxylic acid as a scaffold for developing narrow-spectrum or resistance-targeted antifungal agents [41,42].

Moreover, acridine-4-carboxylic acid exhibited minimal effects on plant growth at lower concentrations, while higher doses led to a marked inhibition of shoot development (Figure 5C,D). A similar trend was observed in the toxicity assessment carried out using the *C. elegans* model (Figure 5B). These results suggest that acridine-4-carboxylic acid is largely non-toxic at sub-inhibitory levels but may present environmental concerns when applied at elevated concentrations. The favorable safety profile at low concentrations holds significant promise as a biofilm-targeting therapeutic agent. Our findings collectively support the selective antifungal potential of the compound while underscoring the need for careful dose optimization in therapeutic or environmental application. While these findings highlight the biological relevance of acridine-4-carboxylic acid, its genotoxicity and mutagenicity potential was not evaluated in this study. Several acridine derivatives are well-documented DNA intercalators [43,44,45]. However, acridine-4-carboxylic acid contains a polar carboxyl group at the 4-position [46], which enhances hydrophilicity and alters electronic distribution, factors known to reduce DNA intercalation and potentially lower genotoxic risk [47]. Genotoxicity in acridine derivatives typically arises from a combination of molecular planarity, charge, and lipophilicity, and even minor structural changes can significantly alter DNA-binding interactions and biological outcomes [44,48,49]. Certain motifs act as genotoxicity amplifiers, such as the 1-nitro group in 1-nitro-9-aminoacridine, which forms DNA adducts and cross-links via bioreduction, a phenomenon not observed in its 2- or 4-nitro isomers. Similarly, the positional orientation of amino groups also influences activity, with 9-aminoacridine known for strong DNA intercalation and mutagenicity [50]. In contrast, pyridoacridine derivatives, which disrupt planarity by fusing a pyridone-like ring to the acridine scaffold, exhibit reduced cytotoxicity and genotoxicity [51]. These observations emphasize the structural-dependent nature of acridine-related genotoxicity. Therefore, future studies involving comprehensive toxicological evaluations are recommended to inform the potential therapeutic development of acridine-4-carboxylic acid.

Moreover, acridine-4-carboxylic acid satisfies key drug-likeness criteria by Lipinski, Veber, and Muegge [52,53], and its physicochemical properties support oral bioavailability with reduced off-target effects (Table 1). Additionally, the absence of predicted interactions with major CYP enzymes and P-glycoprotein suggests a low risk of drug–drug interactions and efflux-mediated resistance. This in silico toxicity assessment further indicates a favorable safety margin [54]. In contrast, other derivatives showed less balanced pharmacokinetics, including excessive lipophilicity and transporter inhibition. Hence, acridine-4-carboxylic acid offers a well-rounded foundation for further development, particularly through structure–activity relationship (SAR) optimization and in vivo validation.

Overall, our findings position acridine-4-carboxylic acid as a promising lead for antifungal drug development, particularly for biofilm-targeted infections. However, due to its moderate toxicity at therapeutic concentrations, further pharmacological optimization such as structural modification or targeted delivery strategies may be necessary to enhance its therapeutic index and clinical applicability.

## 4. Materials and Methods

### 4.1. Candida Strain, Growth Conditions, and Reagents

Two *Candida albicans* strains, DAY185 (fluconazole-resistant) and ATCC 10231 (fluconazole-sensitive), were obtained from the Korean Culture Center of Microorganisms (KCCM) and the American Type Culture Collection (ATCC), respectively. *C. albicans* strains were cultured on potato dextrose agar (PDA) for colony isolation and maintained in potato dextrose broth (PDB) for all experimental procedures. Additional strains such as *Candida glabrata* (ATCC 2001, KCCM 50701), *Candida parapsilosis* (ATCC 7330, KCCM 50030, ATCC 22019), and *Candida auris* (KCTC 17809, KCTC 17810) were sourced from the ATCC, KCCM, or Korean Collection for Type Cultures (KCTC). *C. glabrata* and *C. parapsilosis* strains were grown on PDA for colony formation and cultured in yeast malt (YM) medium supplemented with 2% glucose for MIC determination. *C. auris* strains were similarly grown on PDA for colony formation and maintained in tryptic soy broth (TSB) for MIC testing and biofilm formation assays [14,55]. For all strains, plates were incubated at 37 °C for 48 h to allow colony formation. A distinct colony was then inoculated into 25 mL of potato dextrose broth (PDB) and cultured at 37 °C for 48 h with agitation at 250 rpm for subsequent experiments.

Acridine derivatives and amphotericin B (Figure 1) were purchased from Combi-blocks Inc. (San Diego, CA, USA) and used for antifungal and biofilm inhibitory assessment. All acridine derivatives and amphotericin B were dissolved in dimethyl sulfoxide (DMSO), ensuring that the final DMSO concentration in all working solutions did not exceed 0.1% (*v*/*v*). At this concentration, DMSO had no observable effects on the growth or biofilm development of *C. albicans* DAY185.

### 4.2. Crystal Violet Biofilm Inhibition Assay

The inhibitory effect of acridine derivatives on *C. albicans* biofilm formation was assessed using a modified static microtiter plate method, adapted from previously established protocols [56]. An overnight culture of *C. albicans* DAY185 was diluted in potato dextrose broth (PDB) to a final cell density of ~10^6^ CFU/mL. The compounds were tested at concentrations of 10 and 50 μg/mL. Compounds #4, #7, #8, #11, and #12 were further analyzed in dose-dependent varying concentrations of 0, 5, 10, 20, 50, and 100 μg/mL. Aliquots of 300 μL from each treatment condition were transferred into sterile 96-well flat-bottom polystyrene microplates (SPL Life Sciences, Pocheon-si, Republic of Korea) and incubated at 37 °C for 24 h under static conditions. Following incubation, planktonic cells were removed by gently rinsing three times with distilled water. The adherent biofilms were then stained with 0.1% (*w*/*v*) crystal violet for 20 min at room temperature. Subsequently, excess dye was removed by rinsing with distilled water, and the bound stain was dissolved in 95% ethanol. Absorbance was measured at 570 nm (OD_570_) to quantify biofilm formation.

Cell growth analysis of *C. albicans* treated with acridine-4-carboxylic acid was performed as previously described [57]. A log-phase culture of *C. albicans* (~10^6^ cells/mL) was prepared in Potato Dextrose Broth (PDB) and treated with acridine-4-carboxylic acid at concentrations of 0, 10, 20, 50, and 60 µg/mL. Aliquots of 300 µL were dispensed into a 96-well microtiter plate and incubated statically at 37 °C. The optical density (OD) at 620 nm was measured at two-hour intervals over a 24 h period to monitor planktonic cell growth dynamics under different treatment conditions.

The minimum inhibitory concentration (MIC) and minimum biofilm inhibitory concentration (MBIC) were determined using broth microdilution techniques, as per the Clinical and Laboratory Standards Institute (CLSI) guidelines [58]. All experimental conditions were carried out in triplicate and independently repeated twice to ensure reproducibility.

### 4.3. XTT Assay for Assessing Inhibition of Early-Stage Biofilm Formation

To assess the inhibition of early-stage biofilm formation, the metabolic activity of biofilm cells was evaluated using the XTT reduction assay, following the procedure described by [59], with slight modification. Two-day-old culture of *C. albicans* DAY185 was diluted in potato dextrose broth (PDB) to a final cell density of ~10^6^ CFU/mL and treated with acridine-4-carboxylic acid. Then, 100 µL of the cell suspension was added to the wells of a 96-well microtiter plate and incubated at 37 °C for 4 h at static condition to allow initial biofilm formation. After incubation, wells were gently rinsed with sterile PBS twice to remove non-adherent cells and residual medium. Early-stage biofilm formation was then quantified using the XTT assay. Briefly, 100 µL of freshly prepared solution of XTT and phenazine methosulfate (PMS) in a 50:1 (*v*/*v*) ratio was added to each well and incubated in the dark at 37 °C for 1 h to allow the reduction of XTT to an orange-colored formazan product. The metabolic activity of the biofilm cells was then determined by measuring absorbance at 490 nm using a Multiskan EX microplate reader (Thermo Fisher Scientific, Waltham, MA, USA). Results represent the average of at least three independent cultures.

### 4.4. Preformed Biofilm Dispersal Assay

The ability of acridine-4-carboxylic acid to disperse preformed *C. albicans* biofilms was also evaluated. *C. albicans* DAY185 was diluted in potato dextrose broth (PDB) to a final cell density of approximately 10^6^ CFU/mL and inoculated into 96-well microtiter plates without acridine-4-carboxylic acid. The plate was incubated at 37 °C for 24 h under static conditions to allow biofilm formation. After incubation, planktonic cells were removed by gentle pipetting, and the wells were washed with phosphate-buffered saline (PBS, pH 7.4) to remove non-adherent cells. Fresh PDB containing acridine-4-carboxylic acid at concentrations of none MIC, 2× MIC, and 4× MIC was then added to the wells, followed by an additional 24 h incubation under the same condition. The biofilm biomass was quantified using crystal violet (CV) staining, as described in Section 4.2. Absorbance was measured at 570 nm (OD570) to quantify the extent of biofilm retention or dispersal.

### 4.5. Biofilm Visualization by Live Imaging and SEM

To examine the biofilm inhibitory effects of acridine-4-carboxylic acid, *C. albicans* DAY185 cultures of ~10^6^ cells/mL were inoculated in PDB. A 300 μL aliquot of the culture, containing varying concentrations of acridine-4-carboxylic acid (0, 1, 2, 5, 10, or 20 µg/mL), was transferred into sterile 96-well polystyrene microplates. The plates were incubated at 37 °C for 24 h, without shaking to promote biofilm formation.

After incubation, the wells were gently washed three times with phosphate-buffered saline (PBS, pH 7.4) to remove non-adherent cells. Biofilms remaining on the well surfaces were then visualized using the iRiS™ Digital Cell Imaging System (Logos BioSystems, Anyang, Republic of Korea). Acquired images were processed and reconstructed into both 2D and 3D color-coded biofilm structures using ImageJ software (NIH ImageJ, Bethesda, MD, USA: https://imagej.nih.gov/ij/index.html) accessed on 24 February 2025), as described by [60].

For scanning electron microscopy (SEM), *C. albicans* cultures (~10^6^ cells/mL) were incubated with or without acridine-4-carboxylic acid at concentrations of 0, 1, 2, 5, 10, or 20 µg/mL. A total of 300 μL of aliquot was dispensed into 96-well plates containing sterile nylon filter membranes (0.4 × 0.4 mm^2^). After 24 h incubation at 37 °C, biofilms were fixed with a solution of 2.5% glutaraldehyde and 2% formaldehyde at 4 °C for 24 h.

Fixed biofilms were dehydrated through a graded ethanol series (30%, 50%, 70%, 80%, 95%, and 99%) for 10 min at each step. Dehydrated membranes were subjected to critical-point drying using an HCP-2 device (Hitachi, Tokyo, Japan). The dried specimens were then sputter-coated with platinum and imaged using a Hitachi S-4800 scanning electron microscope operating at 15 kV, following the methodology outlined by [60].

### 4.6. Hyphal Development and Cell Aggregation

The impact of acridine-4-carboxylic acid on hyphal development and cell aggregation in *C. albicans* was assessed following a modified protocol adapted from [61]. Briefly, *C. albicans* DAY185 cells were adjusted to a final concentration of ~10^6^ CFU/mL in potato dextrose broth (PDB), in the presence or absence of acridine-4-carboxylic acid at concentrations of 0, 2, 5, 10, 20, and 50 µg/mL, and incubated at 37 °C in a static condition for 24 h. After incubation, hyphal formation and cell aggregation were visualized using bright-field microscopy with the iRiS™ Digital Cell Imaging System (Logos BioSystems, Anyang, Republic of Korea).

### 4.7. Toxicity Studies of Acridine-4-carboxylic Acid Using Plant and Nematode Models

The phytotoxic potential of acridine-4-carboxylic acid was assessed using a seed germination model, as previously described by [62]. Radish seeds (*Raphanus sativus*) were first surface-sterilized, rinsed thoroughly, and air-dried. For each test concentration, nine seeds were evenly placed on soft agar plates composed of 0.7% agar and 0.86 g/L Murashige and Skoog (MS) basal medium. Acridine-4-carboxylic acid was added to the medium at concentrations of 0, 20, 50, 60, 100, and 200 μg/mL. Plates were incubated under ambient room temperature conditions for 5 days. Seed germination and seedling growth were monitored daily, and stem length was measured to evaluate compound-induced toxicity.

The in vivo cytotoxicity of acridine-4-carboxylic acid was evaluated using *Caenorhabditis elegans* strain fer-15 (b26), as described by [63,64]. Young adult nematodes were synchronized and rinsed twice with M9 buffer and transferred into 96-well plates, with approximately 30 worms per well. Each well contained 300 μL of M9 buffer supplemented with acridine-4-carboxylic acid at concentrations of 0, 2, 10, 20, 50, 60, and 100 μg/mL. Plates were maintained in the dark at 25 °C for 10 days without agitation. Worm viability was assessed by stimulating movement in response to gentle tapping. Live and dead nematodes were counted using the iRiS™ Digital Cell Imaging System (Logos BioSystems, Anyang, Republic of Korea). Results were expressed as the percentage of viable nematodes, and all conditions were tested in triplicate across two independent experiments.

### 4.8. Predictions of Absorption, Distribution, Metabolism, and Excretion (ADME) Properties

The pharmacokinetic and toxicity properties of acridine, acridine-4-carboxylic acid, and 2,7-dibromo-9,9-dimethyl-9,10-dihydroacridine were predicted using computational tools such as preADMET, Molinspiration, and GUSAR, based on data accessed on 23 May 2025. These platforms provided comprehensive evaluations of drug-likeness, absorption, distribution, metabolism, excretion, and toxicity (ADMET) characteristics. These platforms evaluated the bioavailability and other pharmacokinetic properties, including but not limited to Lipinski’s rule of five [65].

## 5. Conclusions

This study demonstrates acridine-4-carboxylic acid is a promising antifungal lead compound with selective antivirulence activity against *C. albicans* DAY185, which is fluconazole-resistant. The compound exhibited potent biofilm inhibitory effects at sub-inhibitory concentrations (≥10 µg/mL), significantly reducing biofilm biomass, hyphal formation, and cell aggregation without impairing planktonic growth. These highlight its potential as a targeted antivirulence agent capable of minimizing selective pressure and subsequent resistance development. Toxicity assessments revealed moderate phytotoxicity and low toxicity in *C. elegans* at therapeutically relevant doses, supporting a favorable safety margin for potential applications. Structurally, the planar aromatic acridine core facilitates membrane permeability, while the carboxylic acid moiety contributes to target engagement via electrostatic interactions with nucleic acids and virulence-related proteins. Taken together, these results position acridine-4-carboxylic acid as a balanced antifungal scaffold with dual benefits of favorable pharmacokinetics and targeted antivirulence action. Future studies should focus on elucidating the molecular targets of acridine-4-carboxylic acid, alongside comprehensive evaluations of its in vivo efficacy and toxicity, to support its potential development for clinical and environmental antifungal applications.

## Figures and Tables

**Table 1:** Absorption, distribution, metabolism, and excretion (ADME) parameters of acridine (#6), acridine-4-carboxylic acid (#7), and 2,7-dibromo-9, 9-dimethyl-9,10-dihydroacridine (#12).

Parameters	Acridine	Acridine-4-carboxylic Acid	2,7-Dibromo-9,9-dimethyl-9,10-dihydroacridine
Lipinski (Pfizer) rule of five	Suitable	Suitable	Suitable
Lipinski rule of five violations	0 violation	0 violation	1 violation
Veber (GSK) rule	Suitable	Suitable	Suitable
Muegge (Bayer) rule	Likely Suitable	Likely Suitable	Likely Suitable
Plasma protein binding	97.8	92.8	98.8
Blood–brain barrier permeability	3.0	2.3	15.1
Lipophilicity (iLOGP)	Not Available	Not Available	Not Available
Water Solubility (Log S)	Moderately Soluble	Moderately Soluble	Poorly Soluble
Pgp Substrate_inhibition	Non-Inhibitor	Non-Inhibitor	Inhibitor
CYP1A2 inhibitor	Non-Inhibitor	Non-Inhibitor	Non-Inhibitor
CYP3A4 inhibitor	Non-Inhibitor	Non-Inhibitor	Non-Inhibitor
Skin_Permeability (logKp, cm/hour)	−2.7	−3.30	−1.5
Gastrointestinal intestinal absorption	Moderate	Low	Low
Caco2	30.3	2.9	55.1
Mouse carcinogenicity	Positive	Positive	Positive
Rat carcinogenicity	Negative	Negative	Negative
Acute algae toxicity	0.10	0.09	0.01
Acute fish toxicity (medaka)	0.02	0.01	0
Acute fish toxicity (minnow)	0.01	0.01	0
In vitro hERG inhibition	Medium_risk	Medium_risk	Medium_risk
miLogP	3.1	2.69	6
Mol volume	167.8	194.8	242.4
TPSA	12.8	50.1	12
GPCR ligand	Inactive	Inactive	Inactive
Ion channel modulator	Inactive	Inactive	Inactive
Kinase inhibitor	Potential Inhibitor	Potential Inhibitor	Inactive
Nuclear receptor ligand	Inactive	Inactive	Inactive
Protease inhibitor	Inactive	Inactive	Inactive
Enzyme inhibitor	Possible	Possible	Inactive
Rat IP LD50 classification	Class IV in AD	Class V in AD	Class V in AD
Rat IV LD50 classification	Class IV in AD	Class IV in AD	Non-Toxic
Rat oral LD50 classification	Class IV in AD	Class IV in AD	Class IV in AD
Rat SC LD50 classification	Class IV in AD	Class V in AD	Class V in AD

## Data Availability

The data utilized in this study can be found within the main text of the article.
